# A prediction model for major adverse cardiovascular events (MACE) in patients with coronavirus disease 2019 (COVID-19)

**DOI:** 10.1186/s12890-022-02143-3

**Published:** 2022-09-12

**Authors:** Dong Huang, Huan Yang, He Yu, Ting Wang, Zhu Chen, Rong Yao, Zongan Liang

**Affiliations:** 1 Department of Respiratory and Critical Care Medicine, West China Hospital, Sichuan University, No 37 Guoxue Alley, Chengdu, 610041 Sichuan China; 2 Department of Infectional Inpatient Ward Two, Chengdu Public Health Clinical Medical Center, Chengdu, Sichuan China; 3 Department of Emergency Medicine, Emergency Medical Laboratory, West China Hospital, Sichuan University, No 37 Guoxue Alley, Chengdu, 610041 Sichuan China; 4 Disaster Medical Center, Sichuan University, Chengdu, Sichuan China

**Keywords:** COVID-19, MACE, Risk factors, Nomogram

## Abstract

**Background:**

Emerging evidence shows that cardiovascular injuries and events in coronavirus disease 2019 (COVID-19) should be considered. The current study was conducted to develop an early prediction model for major adverse cardiovascular events (MACE) during hospitalizations of COVID-19 patients.

**Methods:**

This was a retrospective, multicenter, observational study. Hospitalized COVID-19 patients from Wuhan city, Hubei Province and Sichuan Province, China, between January 14 and March 9, 2020, were randomly divided into a training set (70% of patients) and a testing set (30%). All baseline data were recorded at admission or within 24 h after admission to hospitals. The primary outcome was MACE during hospitalization, including nonfatal myocardial infarction, nonfatal stroke and cardiovascular death. The risk factors were selected by LASSO regression and multivariate logistic regression analysis. The nomogram was assessed by calibration curve and decision curve analysis (DCA).

**Results:**

Ultimately, 1206 adult COVID-19 patients were included. In the training set, 48 (5.7%) patients eventually developed MACE. Six factors associated with MACE were included in the nomogram: age, PaO_2_/FiO_2_ under 300, unconsciousness, lymphocyte counts, neutrophil counts and blood urea nitrogen. The C indices were 0.93 (95% CI 0.90, 0.97) in the training set and 0.81 (95% CI 0.70, 0.93) in the testing set. The calibration curve and DCA demonstrated the good performance of the nomogram.

**Conclusions:**

We developed and validated a nomogram to predict the development of MACE in hospitalized COVID-19 patients. More prospective multicenter studies are needed to confirm our results.

**Supplementary Information:**

The online version contains supplementary material available at 10.1186/s12890-022-02143-3.

## Background

The outbreak of coronavirus disease 2019 (COVID-19), caused by severe acute respiratory syndrome coronavirus 2 (SARS-CoV-2) infections, has been widely recognized and reported since December 2019 [1]. The World Health Organization (WHO) made the assessment that COVID-19 could be characterized as a pandemic in March 2020 [2]. It is still sweeping the globe [3]. The pathogenic mechanism and epidemiological and clinical characteristics of COVID-19 seem not to be very different from those of Severe Acute Respiratory Syndrome (SARS) and Middle East Respiratory Syndrome (MERS) [4]. Therefore, COVID-19 is often considered as a primary respiratory infectious disease and viral pneumonia. However, emerging evidence shows that extrapulmonary manifestations, complications and multiorgan injuries should also be considered [5].

It was reported that the incidences of severe cardiovascular events, such as stroke and acute myocardial infarction, among all COVID-19 patients were 2.5% and 1.1%, respectively [6]. Moreover, they are also significant contributors to the poor prognosis of severe COVID-19 patients. In patients with COVID-19 and ST-segment elevation myocardial infarction (STEMI), 18% of them required mechanical ventilation, 17% required cardiac resuscitation, and 11% eventually died [7]. Another study reported that the mortality of COVID-19 patients with ischemic stroke was approximately 30% [8].

There is little available information about the risk factors and prediction models for major adverse cardiovascular events (MACE), which consist of myocardial infarction, stroke and cardiovascular death. Li et al. [8] reported that older COVID-19 patients with hypertension, diabetes, and high levels of C reactive protein (CRP) and D-dimer were prone to acute cerebrovascular disease. Tan et al. [9] conducted a systemic review and found that elevated D-dimer and fibrinogen, as well as detected antiphospholipid antibodies, were associated with acute ischemic stroke in patients with COVID-19. However, these above conclusions are not identical and need more evaluation because most previous studies were single-center, had limited sample sizes, did not have control groups, or lacked adjustment for confounding factors.

Considering that the severity of disease among different COVID-19 patients is highly heterogeneous, ranging from asymptomatic infection or mild disease to critical illness with multiple organ injuries, early prediction, detection and diagnosis of MACE are of great importance to optimize therapies, which might also improve the prognosis of COVID-19 patients. The current study was conducted to identify related risk factors and to develop and validate an early prediction model for MACE during the hospitalization of COVID-19 patients.

## Methods

### Study design, participants and data collection

This retrospective, multicenter, observational study was conducted on hospitalized patients from two major COVID-19 designated hospitals (Wuhan Red Cross Hospital and People's Hospital of Wuhan University) in Wuhan city, Hubei Province, and 36 COVID-19 designated hospitals, including university teaching hospitals and regional hospitals, in Sichuan Province, China, between January 14 and March 9, 2020. It was conducted in accordance with the amended Declaration of Helsinki and approved by the West China Hospital of Sichuan University Biomedical Research Ethics Committee (No. 2020-272). Written informed consent was waived due to its retrospective observational design. The development and validation of the prediction model were performed in accordance with the TRIPOD (Transparent Reporting of a multivariable prediction model for Individual Prognosis or Diagnosis) statement [10].

For the calculation of sample size, it was estimated that 4–8 variables would eventually be included in the prediction model. In most cases, the events per variable (EPV) should exceed 10 to ensure the accuracy and efficacy of the variables [11]. Therefore, 40–80 patients with MACE were needed. However, up to 10% of patients might drop out or have incomplete clinical data. As a result, 44–88 patients with MACE are warranted. Finally, the incidence of MACE in all COVID-19 patients was less than 10% in previous studies. Thus, the required sample size of COVID-19 patients was estimated to be at least 440–880.

All patients enrolled in this study had laboratory-confirmed COVID-19 according to the WHO interim guidance, which was defined as a positive result for the nucleic acid of SARS-CoV-2 by real-time reverse-transcription polymerase chain reaction (RT‒PCR) [12]. The exclusion criteria were as follows: (1) under 18 years old; (2) being pregnant; (3) died or discharged within 24 h after admission; (4) incomplete clinical data; and (5) recovering from cardiac arrest or cardiopulmonary resuscitation.

The selection of patients was independently completed by two trained clinicians. Any disagreement was resolved by team discussion until a consensus was reached. All included patients were randomly divided into a training set (70% of patients) and a testing set (30%). The training set was applied to develop a prediction model, and the testing set was used to validate the performance of the model. The clinical baseline characteristics between the two sets were compared to ensure similarity or nonsignificant differences.

All clinical data, including demographic characteristics, symptoms, basic vital signs, comorbidities, chest computed tomography (CT) results and laboratory examinations, were collected from the electronic medical records and anonymized. The baseline data were recorded at admission or within 24 h after admission to hospitals. Two experienced doctors independently reviewed the medical records and completed the data collection. Any disagreement was resolved by a third doctor or team discussion until a consensus was reached.

### Study outcomes

The primary outcome was MACE during hospitalization, including nonfatal myocardial infarction, nonfatal stroke and cardiovascular death. This triple composite endpoint is commonly used in studies to investigate the cardiovascular risks of diabetes and the effects of related drugs [13]. It has been used in various cardiovascular studies in recent years.

Specifically, acute myocardial infarction (AMI) was defined as the detection of elevated cardiac troponin with at least 1 value above the 99th percentile upper reference limit and at least one of the following: symptoms of myocardial ischemia; new ischemic electrocardiograph (ECG) changes; imaging evidence of new loss of viable myocardium or new regional wall motion abnormality; and identification of a coronary thrombus by angiography or autopsy [14]. Stroke was diagnosed by its clinical manifestations and confirmed via CT or magnetic resonance imaging (MRI) [15]. Cardiovascular death was defined as death with obvious cardiovascular causes or not definitely attributable to non-cardiovascular causes, including but not limited to fatal myocardial infarction, fatal stroke, sudden death, death caused by cardiogenic shock, death related to cardiovascular procedures and death from other cardiovascular causes.

### Statistical analysis

Data were analyzed with IBM SPSS Statistics version 23.0 and R software 4.0.2. Continuous variables are presented as the mean (standard deviation, SD) or median (interquartile range, IQR) according to their distribution, as determined by the Kolmogorov–Smirnov normality test and Bartlett's test for homogeneity of variance. Categorical variables are shown with counts and percentages. Means for continuous variables were compared by using two-tailed independent Student's *t*-tests when the data were normally distributed; if not, the Mann–Whitney *U* test was used. Proportions for categorical variables were compared with the χ^2^ test or Fisher's exact test.

We used multiple imputation (MI), which was performed by using Bayesian methods in SPSS, to minimize bias due to the retrospective design. It was applied to account for missing data if the missing values were under 20%. The variables with a missing rate of above 20% were excluded. The risk factors were preliminarily selected by LASSO (least absolute shrinkage and selection operator) regression analysis. It was used to minimize the potential collinearity or overfitting of variables measured from the same patient. The LASSO regression model could penalize the regression coefficients of variables through the parameter λ. With the largest penalties and minimum λ, the estimates of weak factors could shrink toward zero and be eliminated. Therefore, the final model only included the strongest predictors. The glmnet package in R was used to perform the LASSO regression.

The patients in the training set were divided into two groups according to the presence or absence of MACE. Variables with *P* < 0.10 were included in the univariate and multivariate logistic regression analyses to identify independent risk factors for MACE. The fitness of multivariate logistic regression model was tested by using a Hosmer–Lemeshow (H–L) test. The interactions and multicollinearity among the independent risk factors were also examined. A variance inflation factors (VIF) > 10 was considered indicative of multicollinearity. We used odds ratios (ORs) and 95% confidence intervals (95% CIs) to evaluate the risk factors.

The prediction model was established through the rms package in R based on the results of LASSO regression and multivariate logistic regression. The goodness of fit of the prediction model was evaluated via the concordance index (C index) with the 95% CIs [16]. Then, a nomogram was visually established based on the prediction model with the highest C index [17]. The nomogram was also assessed by calibration curve and decision curve analysis (DCA) in the training set (internal validation) and testing set (external validation) [18, 19]. After that, subgroup analysis in terms of the regions where COVID-19 patients were from (Wuhan or Sichuan) was also performed to test the performance and accuracy of the model further in different COVID-19 patients. Statistical significance was defined as *P* < 0.05.

## Results

### Baseline patient characteristics

**Fig. 1:**
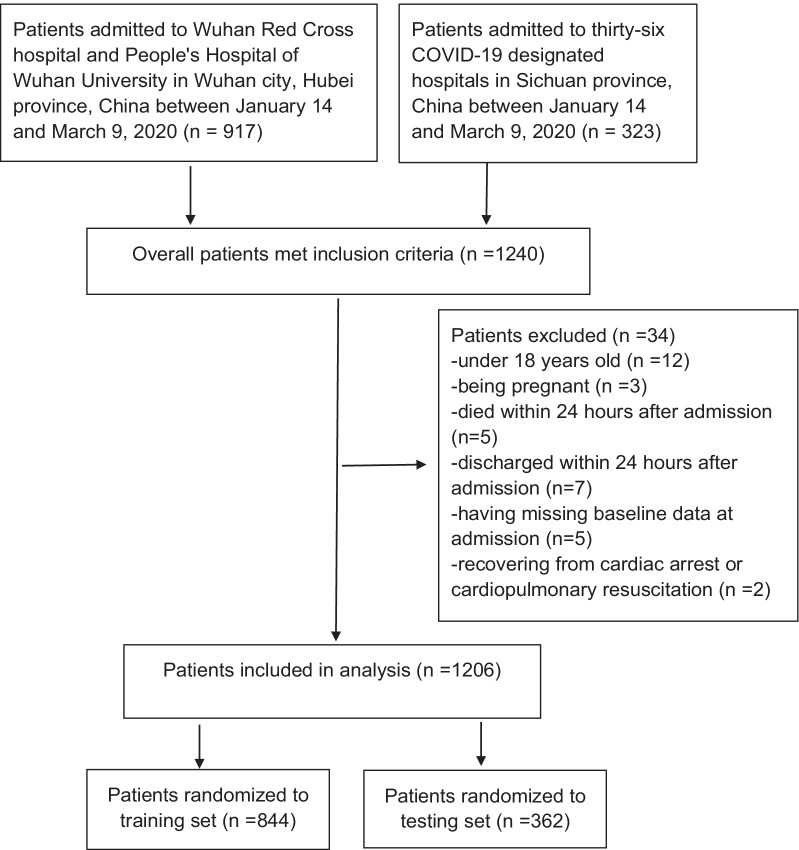
Study population

A total of 1240 patients confirmed to have COVID-19 were included in this study. Finally, 34 of them were excluded after applying the exclusion criteria. Among the remaining 1206 patients, 844 patients (70% of all patients; 633 patients from Wuhan and 211 patients from Sichuan) were randomized to the training set, and 362 patients (30% of all patients; 265 patients from Wuhan and 97 patients from Sichuan) were included in the testing set. The detailed selection of patients is shown in Fig. 1. The clinical baseline characteristics of the training and testing sets are compared in Additional file 1: Table S1. There were a few significant differences in the respiratory rate (*P* = 0.022), chronic neural disease (*P* = 0.008), PT (*P* = 0.042) and Na (*P* = 0.018). Other characteristics were not significantly different between the two sets. Thus, there was no significant bias when splitting the two groups.

In the training set, 48 (5.7%) patients eventually developed MACE. Among them, 8 (0.9%) patients had AMI, and 9 (1%) patients developed stroke. Finally, 38 (4.5%) patients were diagnosed with cardiovascular death. Compared with patients without MACE, the MACE group had a higher proportion of male patients (64.6% vs. 46.9%, *P* = 0.017), median age (72.5 vs. 54 years, *P* < 0.001), respiratory rate (21 vs. 20 breath/min, *P* = 0.005), rate of unconscious patients (16.7% vs. 0.3%, *P* < 0.001), more patients whose PaO_2_/FiO_2_ (arterial partial pressure of oxygen/fraction of inspiration oxygen) was less than 300 (35.4% vs. 10.6%, *P* < 0.001), more comorbidities (chronic heart disease: 22.9% vs. 8.3%, *P* = 0.002; chronic kidney disease: 10.4% vs. 1.3%, *P* = 0.001), etc. Some significant differences in laboratory examination results were also observed between patients with and without MACE. The MACE group had higher neutrophil counts (6.56 × 10^9^/L vs. 3.47 × 10^9^/L, *P* < 0.001), D-dimer (2.29 mg/L vs. 0.57 mg/L, *P* < 0.001), and blood urea nitrogen (BUN, 7.04 mmol/L vs. 4.32 mmol/L, *P* < 0.001) but lower lymphocyte counts (0.54 × 10^9^/L vs. 1.19 × 10^9^/L, *P* < 0.001) and albumin (35.3 g/L vs. 39.1 g/L, *P* < 0.001). Detailed comparisons of the baseline characteristics are shown in Table 1.

**Table 1:** Comparisons of clinical characteristics between patients with MACE and patients without MACE in training set

Variables	Overall (n = 844)	With MACE (n = 48)	Without MACE (n = 796)	*P* value
*Demographic characteristics*				
Sex(male)	404(47.9)	31(64.6)	373(46.9)	0.017
Age, years	55(41,66)	72.5(64,81)	54(39,65)	< 0.001
History of alcohol use	181(21.4)	9(18.8)	172(21.6)	0.639
Smoking history	180(21.3)	11(22.9)	169(21.2)	0.782
*Vital signs on admission*				
Temperature (°C)	36.7(36.4,37)	36.6(36.3,36.9)	36.7(36.4,37.1)	0.657
Heart rate (beat/min)	85(78,96)	84(78,100)	86(78,96)	0.785
Respiratory rate (breath/min)	20(19,21)	21(19,28)	20(19,20)	0.005
Systolic pressure (mmHg)	128(119,140)	130(120,143)	128(119,140)	0.494
Diastolic pressure (mmHg)	79(70,85)	76(66,83)	79(71,85)	0.022
PaO_2_/FiO_2_ under 300	101(12)	17(35.4)	84(10.6)	< 0.001
Glasgow coma scale	15(15,15)	15(14,15)	15(15,15)	< 0.001
*Symptoms and Signs*				
Fever	559(66.2)	35(72.9)	524(65.8)	0.313
Cough	536(63.5)	31(64.6)	505(63.4)	0.873
Hemoptysis	26(3.1)	0(0)	26(3.3)	0.4
Short of breath/dyspnea	176(20.9)	26(54.2)	150(18.8)	< 0.001
Weakness/fatigue	307(36.4)	26(54.2)	281(35.3)	0.008
Sore throat/pharyngalgia	59(7)	1(2.1)	58(7.3)	0.279
Rhinorrhea	18(2.1)	0(0)	18(2.3)	0.59
Wheeze	94(11.1)	12(25)	82(10.3)	0.002
Stuffy nose	13(1.5)	0(0)	13(1.6)	1.00
Chest pain/distress	192(22.7)	14(29.2)	178(22.4)	0.275
Muscle ache/myalgia	84(10)	4(8.3)	80(10.1)	0.891
Arthralgia	15(1.8)	2(4.2)	13(1.6)	0.208
Headache	54(6.4)	1(2.1)	53(6.7)	0.34
Unconsciousness	10(1.2)	8(16.7)	2(0.3)	< 0.001
Stomachache	18(2.1)	2(4.2)	16(2)	0.624
Nausea/vomiting	35(4.1)	2(4.2)	33(4.1)	1.00
Diarrhea	107(12.7)	4(8.3)	103(12.9)	0.352
*Comorbidities*				
Chronic heart disease	77(9.1)	11(22.9)	66(8.3)	0.002
Asthma	5(0.6)	0(0)	5(0.6)	1.00
COPD	22(2.6)	4(8.3)	18(2.3)	0.036
Chronic kidney disease	15(1.8)	5(10.4)	10(1.3)	0.001
Chronic liver disease	53(6.3)	3(6.3)	50(6.3)	1.00
Chronic neural disease	7(0.8)	2(4.2)	5(0.6)	0.055
Cancer	26(3.1)	5(10.4)	21(2.6)	0.009
Diabetes mellitus	120(14.2)	6(12.5)	114(14.3)	0.726
Autoimmune disease	7(0.8)	0(0)	7(0.9)	1.00
Dementia	11(1.3)	2(4.2)	9(1.1)	0.126
Hematological disease	24(2.8)	1(2.1)	23(2.9)	1.00
Stroke history	15(1.8)	2(4.2)	13(1.6)	0.208
Hypertension	232(27.5)	24(50)	208(26.1)	< 0.001
*Laboratory examinations*				
White blood cell, × 10^9^/L	5.5(4.33,7.04)	7.8(5.5,12.5)	5.5(4.3,6.88)	< 0.001
Hemoglobin, g/L	128(118,139)	123.5(108,141)	128(119,139)	0.07
Platelet counts, × 10^9^/L	206(162,259)	200(137,261)	206(163,259)	0.103
Lymphocyte counts, × 10^9^/L	1.19(0.89,1.62)	0.54(0.42,1.15)	1.19(0.92,1.65)	< 0.001
Neutrophil counts, × 10^9^/L	3.5(2.61,4.86)	6.56(4.15,11.18)	3.47(2.56,4.63)	< 0.001
Eosinophils, × 10^9^/L	0.03(0.01,0.08)	0.01(0.01,0.04)	0.03(0.01,0.09)	0.001
Basophils, × 10^9^/L	0.01(0.01,0.03)	0.01(0.01,0.03)	0.01(0.01,0.03)	0.445
Monocyte count, × 10^9^/L	0.43(0.32,0.55)	0.4(0.29,0.50)	0.43(0.32,0.56)	0.381
Hematocrit (%)	0.38(0.35,0.41)	0.36(0.32,0.4)	0.38(0.35,0.41)	0.024
D-dimer, mg/L	0.6(0.37,1.05)	2.29(0.7,9.17)	0.57(0.36,0.88)	< 0.001
Fibrinogen, g/L	3.6(2.85,4.32)	4.83(3.13,6.3)	3.56(2.85,4.24)	0.001
APTT, s	28(26.3,29.7)	28.5(27.4,31.8)	27.8(26.2,29.5)	0.007
PT, s	12(11.5,12.5)	12.8(12,14.1)	12(11.4,12.4)	< 0.001
INR	1.03(0.97,1.06)	1.1(1.03,1.19)	1.03(0.97,1.05)	< 0.001
Total bilirubin, μ mol/L	10.2(8,13.1)	10.6(9.6,19.2)	10.2(7.9,12.8)	0.001
Direct bilirubin, μ mol/L	3.3(2.4,4.4)	4.45(3.3,8.8)	3.3(2.4,4.2)	< 0.001
Indirect bilirubin, μ mol/L	6.9(6,7.5)	6.9(6.9,9.32)	6.9(5.9,7.5)	0.085
ALT, IU/L	24(17,36)	24(18,40)	24(17,36)	0.92
AST, IU/L	24.2(20,33)	29(22,46)	24(20,32)	0.006
Total protein, g/L	64.6(60.1,68.7)	60.5(56.2,64.6)	64.6(60.5,68.9)	< 0.001
Albumin, g/L	39(35.9,42.2)	35.3(31,39.1)	39.1(36.2,42.4)	< 0.001
Globulin, g/L	25.2(22.6,28.1)	25.2(22.2,27.6)	25.2(22.6,28.2)	0.861
Triglyceride, mmol/L	1.23(1.04,1.51)	1.23(1.12,1.65)	1.23(1.03,1.50)	0.163
Cholesterol, mmol/L	4.03(3.62,4.40)	4.04(3.35,4.11)	4.04(3.63,4.44)	0.117
HDL, mmol/L	1(0.92,1.16)	0.94(0.75,1.03)	1.03(0.94,1.16)	0.001
LDL, mmol/L	2.41(2.12,2.65)	2.3(1.94,2.59)	2.41(2.14,2.68)	0.306
CKMB, U/L	1.31(0.99,2.00)	1.83(1.31,4.84)	1.31(0.97,1.75)	0.001
Glucose, mmol/L	5.6(5.01,6.45)	6.14(5.59,8.17)	5.59(4.98,6.38)	< 0.001
Na, mmol/L	141(139,143)	140(138,144)	141(139,143)	0.908
K, mmol/L	4(3.68,4.19)	4.18(3.95,4.62)	3.95(3.66,4.17)	< 0.001
Ca, mmol/L	2.18(2.10,2.27)	2.12(2,2.18)	2.18(2.10,2.28)	< 0.001
Mg, mmol/L	0.85(0.81,0.88)	0.9(0.81,0.96)	0.85(0.81,0.88)	0.248
BUN, mmol/L	4.32(3.60,5.41)	7.04(4.32,12.3)	4.32(3.56,5.24)	< 0.001
Creatinine, μ mol/L	62(52,71)	66.5(59.5,122.5)	62(50,70)	0.001
Uric acid, umol/L	260(215,316)	266(218,399)	260(214,314)	0.271
Myoglobin, g/L	32.3(31.2,33.8)	49.4(32.3,172)	32.3(30.4,32.3)	< 0.001
C-reactive protein, mg/L	10(6.7,15.07)	17.2(10,117)	10(5.94,12.14)	< 0.001
Procalcitonin, μ g/L	0.05(0.04,0.06)	0.11(0.05,0.52)	0.05(0.04,0.05)	< 0.001
*Chest CT scan images*				
Abnormal lobes	4(1,5)	0(0,5)	4(1,5)	< 0.001
Consolidation	135(16)	4(8.3)	131(16.5)	0.136
Ground-glass opacity	662(78.4)	46(95.8)	616(77.4)	0.003
Paving	5(0.6)	0(0)	5(0.6)	1.00
Fibrotic	206(24.4)	5(10.4)	201(25.3)	0.02
Effusion	39(4.6)	4(8.3)	35(4.4)	0.364

Data are shown as median with interquartile range (IQR) for continuous variables or number with percentage for categorical variables

*MACE* major adverse cardiovascular events, *n* numbers, *PaO*_*2*_ arterial partial pressure of oxygen, *FiO*_*2*_ fraction of inspiration oxygen, *COPD* chronic obstructive pulmonary disease, *APTT* activated partial thromboplastin time, *PT* prothrombin time, *INR* international normalized ratio, *ALT* alanine aminotransferase, *AST* aspartate aminotransferase, *HDL* high density lipoprotein, *LDL* low density lipoprotein, *CKMB* creatine kinase-MB, *Na* sodium, *K* potassium, *Ca* calcium, *Mg* magnesium, *BUN* blood urea nitrogen, *CT* computed tomography

### Development of a prediction model for MACE

The above eighty-five variables measured at admission or within 24 h after admission to hospitals were included in the LASSO regression selection. Finally, the LASSO regression model identified 3 variables that were significant predictors of MACE in COVID-19 patients, including unconsciousness, neutrophil counts and BUN (Additional file 1: Fig. S1).

The factors with *P* < 0.1 in Table 1 were included in the univariate and multivariate logistic regression analysis model. The result of H–L test was *P* = 0.333, suggesting good fitness of the multivariate logistic regression analysis model. There were no significant interactions among these factors. All VIF values were around 1, indicating the absence of multicollinearity. The Cox&Snell R^2^ and Nagelkerke R^2^ value was 0.240 and 0.679, respectively. Finally, we found 6 factors that were independently associated with MACE: age, PaO_2_/FiO_2_ under 300, unconsciousness, lymphocyte counts, neutrophil counts and BUN. The detailed ORs and 95%CIs were shown in Table 2.

**Table 2:** The independent risk factors associated with MACE in multivariate logistic regression analysis in training set

Variables	OR	95%CI	*P* value
Age	1.10	1.06, 1.15	< 0.001
PaO_2_/FiO_2_ under 300	8.83	2.76, 28.24	< 0.001
Unconsciousness	46.78	2.99, 730.83	0.006
Lymphocyte counts	0.13	0.04, 0.41	< 0.001
Neutrophil counts	1.17	1.04, 1.32	0.011
BUN	1.11	1.02, 1.21	0.021

*MACE* major adverse cardiovascular events, *OR* odds ratio, *CI* confidence interval, *PaO*_*2*_ arterial partial pressure of oxygen, *FiO*_*2*_ fraction of inspiration oxygen, *BUN* blood urea nitrogen

**Fig. 2:**
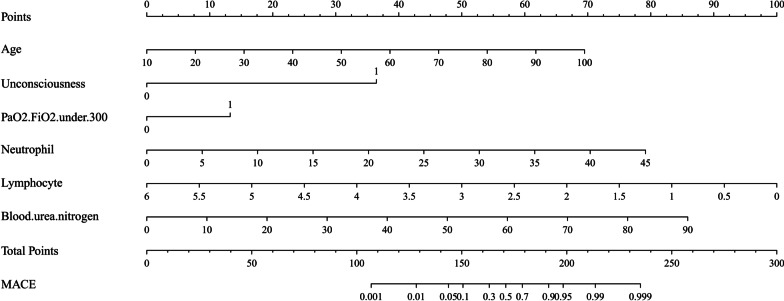
Predictive nomogram for MACE during hospitalizations in COVID-19 patients. Age (years); Unconsciousness and PaO_2_/FiO_2_ (arterial partial pressure of oxygen/fraction of inspiration oxygen) under 300 (1: yes, 0: no); Neutrophil and Lymphocyte: × 10^9^/L; Blood urea nitrogen: mmol/L; MACE major adverse cardiovascular events

As a result, these six factors were included in the prediction model, as described in Fig. 2. Each predictive factor was assigned a single score, which is presented on the top line of the nomogram. The total score for each patient is the sum of each single score. At the bottom of the nomogram, the probabilities of MACE during hospitalization in patients with COVID-19 were predicted in terms of the total scores.

### Validation of the prediction model for MACE

**Fig. 3:**
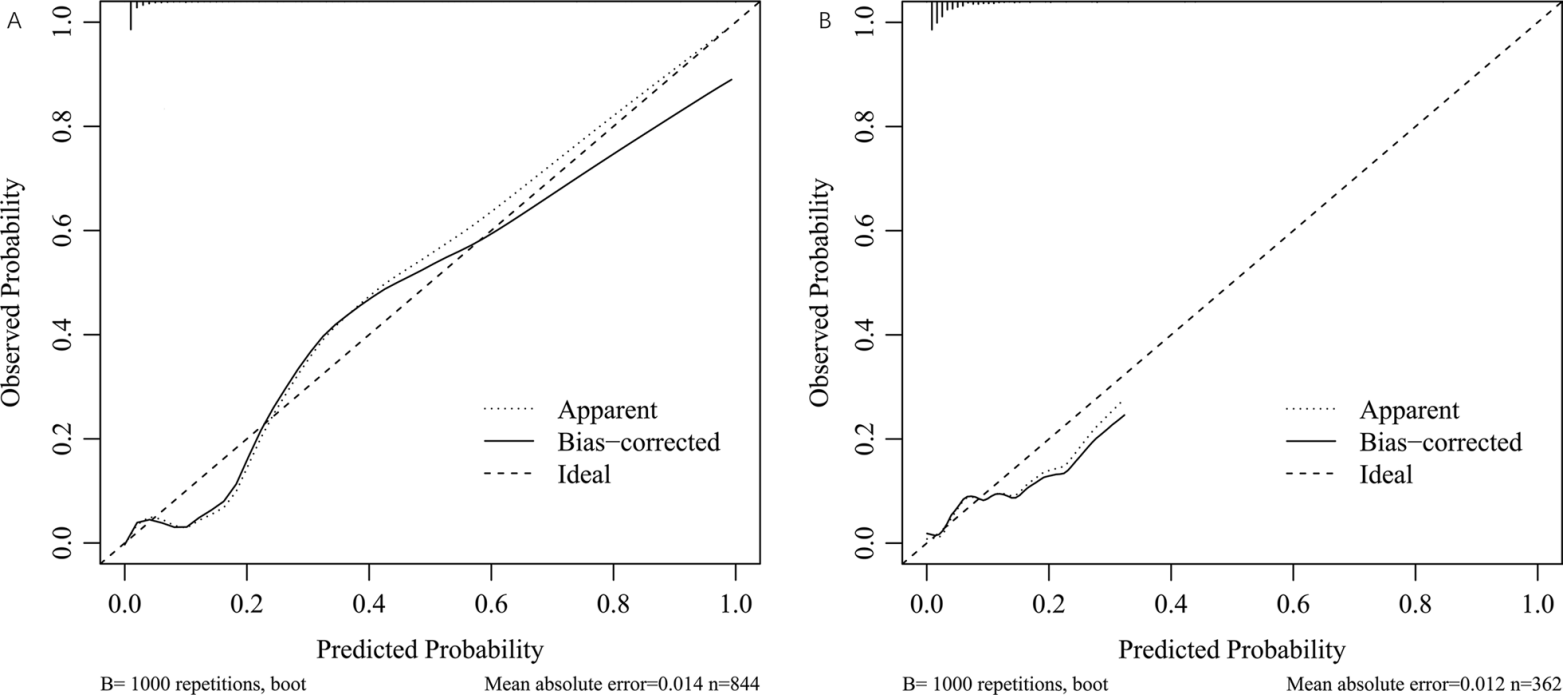
**A** The calibration curve of nomogram in training set. **B** The calibration curve of nomogram in testing set

**Fig. 4:**
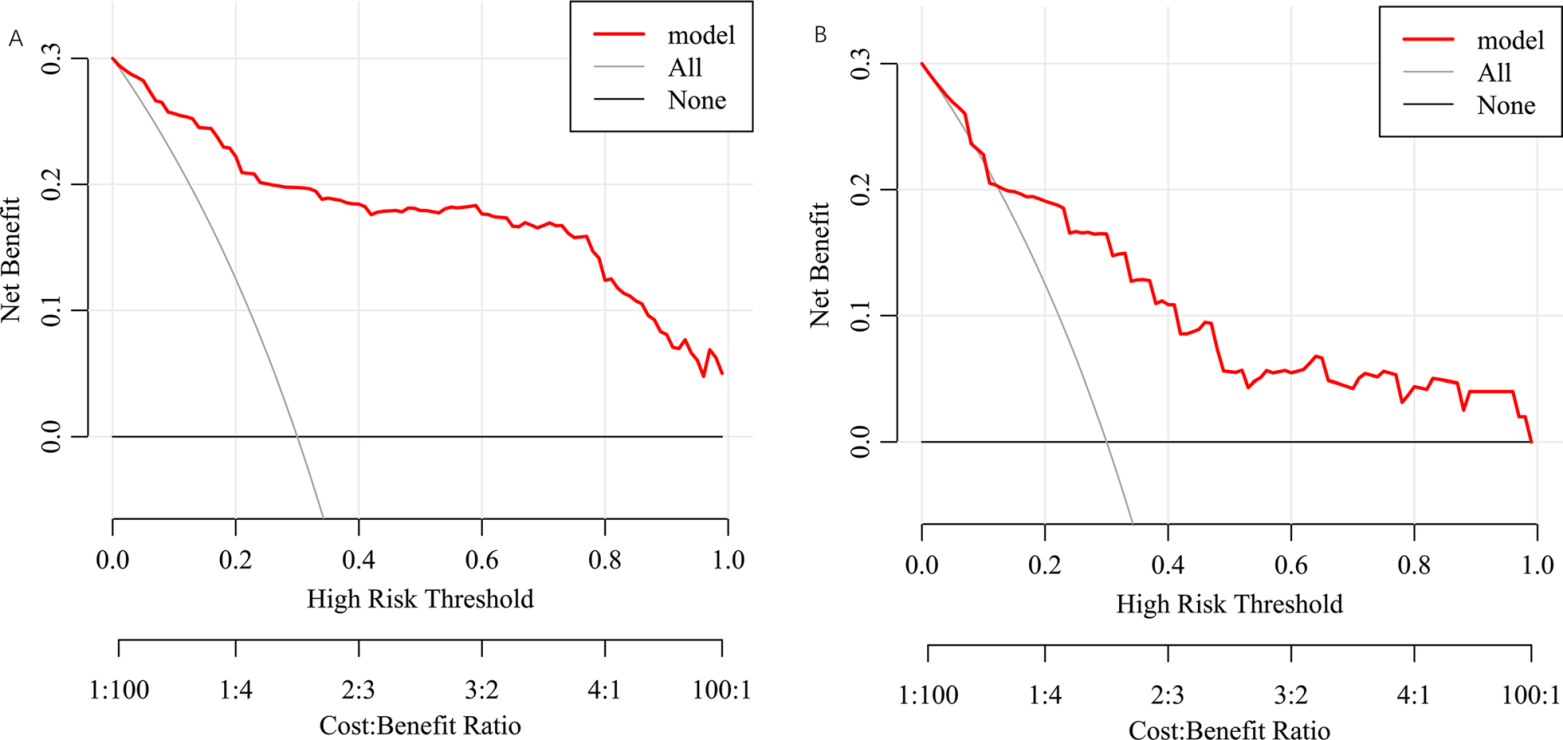
**A** The DCA of nomogram in training set. **B** The DCA of nomogram in testing set. *DCA* decision curve analysis

By using the bootstrap method, the C index was 0.93 (95% CI 0.90, 0.97) in the training set, which indicated that the predictive value was good. As shown in Fig. 3A, the calibration curve did not deviate from the reference line significantly. There was good consistency between the values predicted by the nomogram and the actual observed values in the training set. The adjusted C index was 0.93. To evaluate the clinical applicability of the prediction model, DCA was performed, as shown in Fig. 4A. DCA demonstrated that the nomogram had good overall net benefits within a wide range of threshold probabilities. The nomogram could improve patient outcomes in clinical practice.

In the testing set, 15 (4.1%) patients were diagnosed with MACE. Specifically, 6 (1.7%) patients developed AMI, 2 (0.6%) patients developed stroke, and 10 (2.8%) patients were diagnosed with cardiovascular death. The C index was 0.81 (95% CI 0.70, 0.93) in the testing set, which was lower than that in the training set. As shown in Fig. 3B, the calibration curve indicated good agreement between the estimated results and the actual results of MACE. The adjusted C index was 0.75. The prediction model had stability and external validity to some degree. Figure 4B shows the DCA of the nomogram in the testing set. Similarly, the net benefits within a wide range of threshold probabilities showed that the nomogram had positive impacts on patient outcomes.

In the subgroup analysis, a total of 57 (6.3%) COVID-19 patients had MACE (AMI: 11 [1.2%]; stroke: 9 [1.0%]; cardiovascular death: 45 [5.0%]), and the C index was 0.89 (95% CI 0.84, 0.94) in the Wuhan subgroup. The calibration curve and DCA are shown in Additional file 1: Figs. S2 and S3 in the supplementary material. The adjusted C index was 0.88. In the Sichuan subgroup, 6 (1.9%) patients had MACE (AMI: 3 [1%]; stroke: 2 [0.6%]; cardiovascular death: 3 [1%]). The C index was 0.97 (95% CI 0.92, 1.02), and the adjusted C index was 0.96. The calibration curve and DCA are shown in Additional file 1: Figs. S4 and S5 in the supplementary material. The results of the subgroup analysis were similar to those of the overall analysis.

## Discussion

In the present study, we included over 1200 confirmed adult COVID-19 patients from Wuhan, a relatively high prevalence area, and Sichuan, a relatively low prevalence area, treated at the beginning of 2020 to ensure the accuracy and applicability of the results. The nomogram comprehensively included various aspects of COVID-19 pathophysiology, including demographic characteristics, symptoms, vital signs and laboratory results. Its performance and accuracy were satisfactory based on the C index, which varied from 0.814 to 0.970 in all sets and subgroups. The calibration curve and DCA verified its clinical value.

The pathophysiological mechanisms of cardiovascular injuries or events in COVID-19 are still uncertain thus far and have varied across previous studies. Limited data show that potential explanations include direct viral toxicity and myocyte injury through the angiotensin-converting enzyme 2 (ACE2) receptor in host cells, dysregulation of the renin–angiotensin–aldosterone system (RAAS), endothelial cell damage, immune-mediated cytokine storm syndrome or stress-related cardiomyopathy, a mismatch between oxygen supply and demand, hypercoagulable state and dysregulation of fibrinolysis and thrombosis [20–23]. Nevertheless, these different pathways and roles of various cytokines and other components in the development of cardiovascular events remain an area of active investigation.

Advanced age has been generally known as a risk factor for critical illness or a poor prognosis of COVID-19 in a series of prior studies [24, 25]. However, the other variables in the nomogram have been relatively less explored. We should be more cautious about using them in clinical practice. The presence of decreased PaO_2_/FiO_2_ and unconsciousness indicate that these patients are already seriously ill or critically ill at admission. As a result, their incidences of cardiovascular events, multiple organ injuries and other adverse clinical outcomes are all comparatively high. A systemic review showed that age (per 10 years increase OR 1.8, 95% CI 1.54–2.1; certainty of the evidence: high), hypoxemia (OR 5.46, 95% CI 2.05–14.53; certainty of the evidence: moderate), decreased lymphocytes (OR 3.57, 95% CI 2–6.67; certainty of the evidence: moderate), increased neutrophils (OR 6.78, 95% CI 3.07–14.97; certainty of the evidence: low) and high BUN (OR 10.56, 95% CI 6.76–16.48; certainty of the evidence: low) were all prognostic factors for mortality. Lymphocytes, neutrophils and BUN were additional predictive factors for severe COVID-19 [26]. SARS-CoV-2 has the potential to invade both the central and peripheral nervous systems. It enters the brain via the hematogenous route or the olfactory system. Severe neurological manifestations, including agitation, delirium, and coma, are probably due to hypoxic and metabolic abnormalities [27]. Additionally, two prior studies with large sample sizes revealed that in COVID-19 patients, unconsciousness (OR, 4.71; 95% CI 1.39–15.98) was an independent predictive factor of critical illness (admission to the intensive care unit or invasive ventilation or death) [28], and peripheral oxygen saturation under 92% (HR 2.12, CI 1.56–2.88) was associated with an increased risk of in-hospital mortality [29].

Previous preclinical and clinical studies have well explored the predictive and prognostic values of laboratory results, which agree with our results. Lymphocytopenia, which is caused by systemic inflammation and immunocompromised status, has been considered one of the most common clinical characteristics of COVID-19. Lymphocytes play a crucial role in the modulation of the systemic inflammatory response and atherosclerotic process. Furthermore, lymphocytopenia is related to accelerated atherosclerosis, acute coronary syndromes and a poor prognosis of patients [30]. It has been reported that a high neutrophil to lymphocyte ratio (NLR) has good sensitivity and specificity for a COVID-19 diagnosis in all patients admitted to hospitals [31]. Another meta-analysis of 17 articles with 3396 COVID-19 patients showed that a significant decrease in lymphocytes and increases in neutrophils and BUN were observed in severe COVID-19 patients compared with non-severe patients [32]. The impacts of BUN, a biomarker of kidney injury, on cardiovascular events are still unclear. In 2009 influenza A (H1N1) viral pneumonia, compared with patients without acute kidney injury (AKI), patients with AKI presented more marked cardiovascular, respiratory, and hematological dysfunction [33]. In COVID-19, AKI was a predictor of fatality (OR 14.63, 95% CI 9.94–21.51) and severe infection (OR 8.11, 95% CI 5.01–13.13). A higher level of serum BUN was also associated with a significant increase in fatality (mean difference, MD: 4.07 mmol/L, 95% CI 3.33–4.81) and severe infection (MD: 2.12 mmol/L, 95% CI 1.74–2.50) [34].

The development of MACE in COVID-19 patients has also been reported. Pareek et al. included 586 COVID-19-positive patients and found that higher respiratory rates, altered mental status, and higher troponin T could predict MACE, which was defined as a composite of myocardial infarction, stroke, new acute decompensated heart failure, venous thromboembolism, ventricular or atrial arrhythmia, pericardial effusion, or aborted cardiac arrest. The incidence of MACE in their cohort was 23.0% [35]. In another study from Henein et al., among the 748 patients included, 141 (19%) reached the set endpoint of MACE, a composite of in-hospital CV death, acute heart failure (AHF), acute myocarditis, arrhythmias, acute coronary syndromes (ACS), cardiocirculatory arrest, and pulmonary embolism (PE). In multivariate analysis, troponin and renal failure were good predictors [36]. In another cohort of 839 patients with COVID-19, 72 (9%) patients had MACE, defined as CV death, heart failure, myocardial infarction, nonfatal stroke and major arrhythmia. A history of CVD, age, male sex, chronic obstructive pulmonary disease and lung infiltration on CT were associated with MACE [OR = 2.4; (95% CI 1.6–3.5)] [37]. The incidence of MACE in our study was significantly lower than in the above studies. In the present study, the definition of MACE only represents the three most severe cardiovascular events, which are considered to be associated with the worst prognosis of COVID-19 patients to some degree. Therefore, our study might have some clinical necessity and significance. One of the advantages of the current study is that we provide robust evidence about the relationships between these variables and the probability of MACE in COVID-19 patients. Meanwhile, to the best of our knowledge, we conducted and validated a nomogram for MACE for the first time. It has good clinical utility and reliability.

The current study aimed to explore the possible relationships between these variables and cardiovascular events. Nevertheless, due to the uncertain roles these risk factors play in the development of cardiovascular events and their underlying pathophysiological features, they should be considered and explored in future studies, which might provide more mechanistic and therapeutic insights. Tools to identify patients who are at risk of various complications and a poor prognosis are sorely needed, especially in a resource-limited setting. In the nomogram, the variables are all relatively inexpensive to measure. The clinical characteristics are readily available within minutes, and the laboratory findings are easily obtained within hours. Thus, this nomogram could be used by physicians to predict the likelihood of MACE in hospitalized COVID-19 patients. High-risk patients might need earlier aggressive and individualized monitors, support and treatments, which are crucial for improving their prognosis. There were some differences in the C index in different sets and areas. The heterogeneities in the characteristics of the population and incidence of MACE might be partly responsible for these results. It should be noted that the incidence of MACE in Sichuan Province was low. In addition, our nomogram was developed and based on data collected at the beginning of 2020, during the 1st wave of the COVID-19 pandemic with the initial COVID-19 variant. Recently, variants of COVID-19 have been shown to be less virulent and the vaccination rates are increased around the world. As a result, the probability of MACE in COVID-19 patients now is significantly lower than that in 2020. We admit that the predictive accuracy of nomogram could probably be decreased. However, we believe that the associations of these identified risk factors with development of MACE still exist in COVID-19 patients. Thus, our conclusions remain useful for decision making in clinical practice at present. However, considering the retrospective design, a small minority of incomplete baseline data and the heterogeneity of the composite endpoints, more large-scale, multicenter studies in different epidemiological or environmental situations and genetic backgrounds are warranted to validate or update the prediction model before it can be used in clinical practice.

This study has several limitations. First, our study had unavoidable selection bias due to its retrospective observational nature. The sample size and number of patients with MACE were limited. We only included patients from two provinces in China without further external validation. Although our results have been validated in a distinct cohort, it is acknowledged that the model was derived from particular periods and places and it need to be updated or recalibrated as time goes on. Second, the results might have been affected by some confounders, including the drugs and therapies given before admission, the cardiovascular and renal comorbidities, and the heterogeneity of treatments among the different medical centers, which was especially significant at the beginning of the epidemic. We have tried to adjust for confounding factors in the multivariate analysis as much as possible, but our results still need more validation in the future. Third, we failed to conduct follow-ups or record cardiovascular events after discharge due to the scarcity of relative information. The long-term cardiovascular injuries caused by COVID-19 remain to be studied.

## Conclusions

We developed and validated a nomogram that incorporated age, PaO_2_/FiO_2_ under 300, unconsciousness, lymphocyte counts, neutrophil counts and BUN to predict the development of MACE in hospitalized COVID-19 patients. The results of the C index, calibration curve and DCA showed the good performance and utility of the nomogram. It could empower front-line healthcare providers, inform decision-making, direct healthcare resources and improve patient prognosis. More prospective multicenter studies are needed to confirm our results.

## Supplementary Information

**Additional file 1.** **Table S1.** Comparisons of clinical baseline characteristics between training set and testing set. **Figure S1.** LASSO regression selection for MACE in training set. **Figure S2.** The calibration curve of nomogram in Wuhan subgroup. **Figure S3.** The DCA of nomogram in Wuhan subgroup. **Figure S4.** The calibration curve of nomogram in Sichuan subgroup. **Figure S5.** The DCA of nomogram in Sichuan subgroup.

## Data Availability

The datasets used and/or analysed during the current study are available from the corresponding author on reasonable request.

## Abbreviations

MACE: Major adverse cardiovascular events
COVID-19: Coronavirus disease 2019
DCA: Decision curve analysis
BUN: Blood urea nitrogen
SARS-CoV-2: Severe acute respiratory syndrome coronavirus 2
WHO: World Health Organization
SARS: Severe acute respiratory syndrome
MERS: Middle East respiratory syndrome
PT: Prothrombin time
STEMI: ST-segment elevation myocardial infarction
CRP: C reactive protein
EPV: Events per variable
RT-PCR: Reverse-transcription polymerase chain reaction
CT: Computed tomography
AMI: Acute myocardial infarction
MRI: Magnetic resonance imaging
SD: Standard deviation
IQR: Interquartile range
LASSO: Least absolute shrinkage and selection operator
OR: Odds ratio
95% CI: 95% Confidence interval
PaO_2_/FiO_2_: Arterial partial pressure of oxygen/fraction of inspiration oxygen
ACEII: Angiotensin-converting enzyme type II
RAAS: Renin–angiotensin–aldosterone system
HR: Hazard ratio
NLR: Neutrophil lymphocyte ratio
AKI: Acute kidney injury
MD: Mean difference
NT-proBNP: N-terminal pro brain natriuretic peptide
MIS-C: Multisystem inflammatory syndrome in children
