# Supplementary material for: A prediction model for major adverse cardiovascular events (MACE) in patients with coronavirus disease 2019 (COVID-19)
Source: BMC Pulm Med. 2022 Sep 12;22:343. doi: 10.1186/s12890-022-02143-3 (PMC9466355; doi:10.1186/s12890-022-02143-3)
Supplement: Supplementary file 1 — Additional file 1. Table S1. Comparisons of clinical baseline characteristics between training set and testing set. Figure S1. LASSO regression selection for MACE in training set. Figure S2. The calibration curve of nomogram in Wuhan subgroup. Figure S3. The DCA of nomogram in Wuhan subgroup. Figure S4. The calibration curve of nomogram in Sichuan subgroup. Figure S5. The DCA of nomogram in Sichuan subgroup. [file 12890_2022_2143_MOESM1_ESM.docx]

Table S1. Comparisons of clinical baseline characteristics between training set and testing set.

| Variables | Overall (n=1206) | training set (n=844) | testing set (n=362) | P value |
| --- | --- | --- | --- | --- |
| Demographic characteristics |  |  |  |  |
| Sex(male) | 569 (47.2) | 404 (47.9) | 165 (45.6) | 0.505 |
| Age, years | 55.00 (41.00, 66.00) | 55.00 (41.00, 66.00) | 55.00 (41.00, 66.00) | 0.94 |
| History of alcohol use | 251 (20.8) | 181 (21.4) | 70 (19.3) | 0.454 |
| Smoking history | 251 (20.8) | 180 (21.3) | 71 (19.6) | 0.552 |
| Vital signs on admission |  |  |  |  |
| Temperature (℃) | 36.70 (36.40, 37.00) | 36.70 (36.40, 37.00) | 36.60 (36.40, 37.00) | 0.971 |
| Heart rate (beat/min) | 85.00 (78.00, 96.00) | 85.00 (78.00, 96.00) | 85.00 (78.00, 96.00) | 0.927 |
| Respiratory rate (breath/min) | 20.00 (19.00, 21.00) | 20.00 (19.00, 21.00) | 20.00 (19.00, 22.00) | 0.022 |
| Systolic pressure (mmHg) | 127.00 (119.00, 139.00) | 128.00 (119.00, 140.00) | 126.00 (118.00, 136.00) | 0.105 |
| Diastolic pressure (mmHg) | 79.00 (71.00, 85.00) | 79.00 (70.00, 85.00) | 79.00 (71.25, 85.00) | 0.663 |
| PaO_2_/FiO_2_ under 300 | 146 (12.1) | 101 (12.0) | 45 (12.4) | 0.896 |
| Glasgow coma scale | 15.00 (15.00, 15.00) | 15.00 (15.00, 15.00) | 15.00 (15.00, 15.00) | 0.473 |
| Symptoms and Signs |  |  |  |  |
| Fever | 790 (65.5) | 559 (66.2) | 231 (63.8) | 0.457 |
| Cough | 769 (63.8) | 536 (63.5) | 233 (64.4) | 0.827 |
| Hemoptysis | 36 (3.0) | 26 (3.1) | 10 (2.8) | 0.91 |
| Short of breath/dyspnea | 267 (22.1) | 176 (20.9) | 91 (25.1) | 0.117 |
| Weakness/Fatigue | 437 (36.2) | 307 (36.4) | 130 (35.9) | 0.93 |
| Sore throat/pharyngalgia | 94 (7.8) | 59 (7.0) | 35 (9.7) | 0.141 |
| Rhinorrhea | 28 (2.3) | 18 (2.1) | 10 (2.8) | 0.648 |
| Wheeze | 138 (11.4) | 94 (11.1) | 44 (12.2) | 0.682 |
| Stuffy nose | 22 (1.8) | 13 (1.5) | 9 (2.5) | 0.373 |
| Chest pain/distress | 269 (22.3) | 192 (22.7) | 77 (21.3) | 0.624 |
| Muscle ache/Myalgia | 107 (8.9) | 84 (10.0) | 23 (6.4) | 0.057 |
| arthralgia | 21 (1.7) | 15 (1.8) | 6 (1.7) | 1 |
| Headache | 68 (5.6) | 54 (6.4) | 14 (3.9) | 0.107 |
| Unconsciousness | 13 (1.1) | 10 (1.2) | 3 (0.8) | 0.807 |
| stomachache | 22 (1.8) | 18 (2.1) | 4 (1.1) | 0.323 |
| Nausea/Vomiting | 45 (3.7) | 35 (4.1) | 10 (2.8) | 0.319 |
| Diarrhea | 142 (11.8) | 107 (12.7) | 35 (9.7) | 0.165 |
| Comorbidities |  |  |  |  |
| Chronic heart disease | 109 (9.0) | 77 (9.1) | 32 (8.8) | 0.962 |
| Asthma | 9 (0.7) | 5 (0.6) | 4 (1.1) | 0.56 |
| COPD | 37 (3.1) | 22 (2.6) | 15 (4.1) | 0.216 |
| Chronic kidney disease | 21 (1.7) | 15 (1.8) | 6 (1.7) | 1 |
| Chronic liver disease | 76 (6.3) | 53 (6.3) | 23 (6.4) | 1 |
| chronic neural disease | 18 (1.5) | 7 (0.8) | 11 (3.0) | 0.008 |
| cancer | 35 (2.9) | 26 (3.1) | 9 (2.5) | 0.707 |
| Diabetes mellitus | 176 (14.6) | 120 (14.2) | 56 (15.5) | 0.635 |
| autoimmune disease | 14 (1.2) | 7 (0.8) | 7 (1.9) | 0.178 |
| dementia | 18 (1.5) | 11 (1.3) | 7 (1.9) | 0.57 |
| Hematological disease | 31 (2.6) | 24 (2.8) | 7 (1.9) | 0.474 |
| stroke history | 24 (2.0) | 15 (1.8) | 9 (2.5) | 0.56 |
| Hypertension | 336 (27.9) | 232 (27.5) | 104 (28.7) | 0.711 |
| Laboratory examinations |  |  |  |  |
| White blood cell, ×10^9^/L | 5.50 (4.34, 6.96) | 5.50 (4.33, 7.04) | 5.50 (4.37, 6.80) | 0.592 |
| Hemoglobin, g/L | 128.00 (118.00, 139.00) | 128.00 (118.00, 139.00) | 128.00 (117.00, 138.00) | 0.814 |
| Platelet counts, ×10^9^/L | 206.00 (161.25, 262.75) | 206.00 (162.00, 259.00) | 206.00 (159.25, 266.00) | 0.701 |
| Lymphocyte counts, ×10^9^/L | 1.19 (0.88, 1.63) | 1.19 (0.89, 1.62) | 1.19 (0.87, 1.65) | 0.998 |
| Neutrophil counts, ×10^9^/L | 3.47 (2.59, 4.83) | 3.5(2.61,4.86) | 3.47 (2.55, 4.62) | 0.77 |
| Eosinophils, ×10^9^/L | 0.03 (0.00, 0.08) | 0.03 (0.01, 0.08) | 0.03 (0.00, 0.08) | 0.724 |
| Basophils, ×10^9^/L | 0.01 (0.01, 0.03) | 0.01 (0.01, 0.03) | 0.01 (0.01, 0.02) | 0.446 |
| Monocyte count, ×10^9^/L | 0.43 (0.32, 0.55) | 0.43 (0.32, 0.55) | 0.43 (0.32, 0.54) | 0.202 |
| Hematocrit (%) | 0.38 (0.35, 0.41) | 0.38 (0.35, 0.41) | 0.38 (0.35, 0.41) | 0.47 |
| D-dimer, mg/L | 0.57 (0.37, 1.03) | 0.6(0.37,1.05) | 0.57 (0.35, 1.00) | 0.582 |
| Fibrinogen, g/L | 3.56 (2.92, 4.34) | 3.6(2.85,4.32) | 3.56 (2.99, 4.42) | 0.615 |
| APTT, s | 27.80 (26.30, 29.70) | 28(26.3,29.7) | 27.80 (26.33, 29.70) | 0.758 |
| PT, s | 12.00 (11.60, 12.60) | 12.00 (11.50, 12.50) | 12.00 (11.80, 12.60) | 0.042 |
| INR | 1.03 (0.98, 1.06) | 1.03 (0.97, 1.06) | 1.03 (1.00, 1.06) | 0.146 |
| Total bilirubin, μ mol/L | 10.20 (8.10, 13.00) | 10.2(8,13.1) | 10.20 (8.40, 12.88) | 0.426 |
| Direct bilirubin, μ mol/L | 3.30 (2.40, 4.30) | 3.30 (2.40, 4.40) | 3.30 (2.52, 4.21) | 0.679 |
| Indirect bilirubin, μ mol/L | 6.90 (6.30, 7.60) | 6.90 (6.00, 7.50) | 6.90 (6.80, 8.00) | 0.056 |
| ALT, IU/L | 24.00 (17.00, 36.00) | 24.00 (17.00, 36.00) | 24.00 (17.00, 35.00) | 0.743 |
| AST, IU/L | 24.20 (20.00, 33.00) | 24.20 (20.00, 33.00) | 24.20 (21.00, 32.67) | 0.668 |
| Total protein, g/L | 64.60 (60.50, 68.60) | 64.6(60.1,68.7) | 64.60 (61.35, 68.30) | 0.353 |
| Albumin, g/L | 39.10 (35.90, 42.20) | 39(35.9,42.2) | 39.10 (36.02, 42.20) | 0.747 |
| Globulin, g/L | 25.20 (22.80, 28.20) | 25.20 (22.60, 28.10) | 25.20 (23.10, 28.48) | 0.763 |
| Triglyceride, mmol/L | 1.23 (1.08, 1.51) | 1.23(1.04,1.51) | 1.23 (1.12, 1.50) | 0.527 |
| Cholesterol, mmol/L | 4.04 (3.68, 4.40) | 4.03(3.62,4.40) | 4.04 (3.84, 4.41) | 0.145 |
| HDL, mmol/L | 1.03 (0.93, 1.15) | 1(0.92,1.16) | 1.03 (0.94, 1.12) | 0.79 |
| LDL, mmol/L | 2.41 (2.18, 2.65) | 2.41(2.12,2.65) | 2.41 (2.23, 2.66) | 0.511 |
| CKMB, U/L | 1.31 (1.03, 2.02) | 1.31 (0.99, 2.00) | 1.31 (1.10, 2.12) | 0.217 |
| Glucose, mmol/L | 5.59 (5.05, 6.50) | 5.6(5.01,6.45) | 5.59 (5.12, 6.68) | 0.206 |
| Na, mmol/L | 141.00 (139.00, 143.00) | 141.00 (139.00, 143.00) | 141.00 (138.02, 143.00) | 0.018 |
| K, mmol/L | 3.95 (3.67, 4.21) | 4(3.68,4.19) | 3.95 (3.64, 4.26) | 0.958 |
| Ca, mmol/L | 2.18 (2.10, 2.28) | 2.18 (2.10, 2.27) | 2.18 (2.10, 2.28) | 0.437 |
| Mg, mmol/L | 0.85 (0.82, 0.88) | 0.85 (0.81, 0.88) | 0.85 (0.83, 0.87) | 0.764 |
| BUN, mmol/L | 4.32 (3.60, 5.40) | 4.32(3.60,5.41) | 4.32 (3.50, 5.24) | 0.176 |
| Creatinine, μ mol/L | 62.00 (52.00, 71.75) | 62.00 (52.00, 71.00) | 62.00 (52.00, 72.22) | 0.281 |
| Uric acid, umol/L | 260.00 (217.25, 317.75) | 260.00 (215.00, 316.00) | 260.00 (220.00, 321.02) | 0.488 |
| Myoglobin, g/L | 32.28 (31.49, 33.48) | 32.3(31.2,33.8) | 32.28 (32.26, 32.59) | 0.938 |
| C-reactive protein, mg/L | 10.00 (7.10, 15.49) | 10.00 (6.70, 15.07) | 10.00 (7.46, 19.35) | 0.689 |
| Procalcitonin, μ g/L | 0.05 (0.04, 0.06) | 0.05 (0.04, 0.06) | 0.05 (0.04, 0.05) | 0.926 |
| Chest CT scan images |  |  |  |  |
| Abnormal lobes | 4.00 (1.00, 5.00) | 4.00 (1.00, 5.00) | 4.00 (1.00, 5.00) | 0.345 |
| Consolidation | 197 (16.3) | 135 (16.0) | 62 (17.1) | 0.687 |
| Ground-glass opacity | 935 (77.5) | 662 (78.4) | 273 (75.4) | 0.281 |
| Paving | 7 (0.6) | 5 (0.6) | 2 (0.6) | 1 |
| Fibrotic | 298 (24.7) | 206 (24.4) | 92 (25.4) | 0.765 |
| Effusion | 53 (4.4) | 39 (4.6) | 14 (3.9) | 0.666 |
| Incidence of MACE (%) | 63 (5.2) | 48 (5.7) | 15 (4.1) | 0.336 |

Data are shown as median with interquartile range (IQR) for continuous variables or number with percentage for categorical variables.

n: numbers; PaO_2_: arterial partial pressure of oxygen; FiO_2_: Fraction of inspiration oxygen; COPD, Chronic Obstructive Pulmonary Disease; APTT: Activated partial thromboplastin time; PT: Prothrombin time; INR: international normalized ratio; ALT: Alanine aminotransferase; AST: Aspartate aminotransferase; HDL: high density lipoprotein; LDL: low density lipoprotein; CKMB: creatine kinase-MB; Na: sodium; K: potassium; Ca: calcium; Mg: magnesium; BUN: Blood urea nitrogen; CT: computed tomography; MACE：Major Adverse Cardiovascular Events.


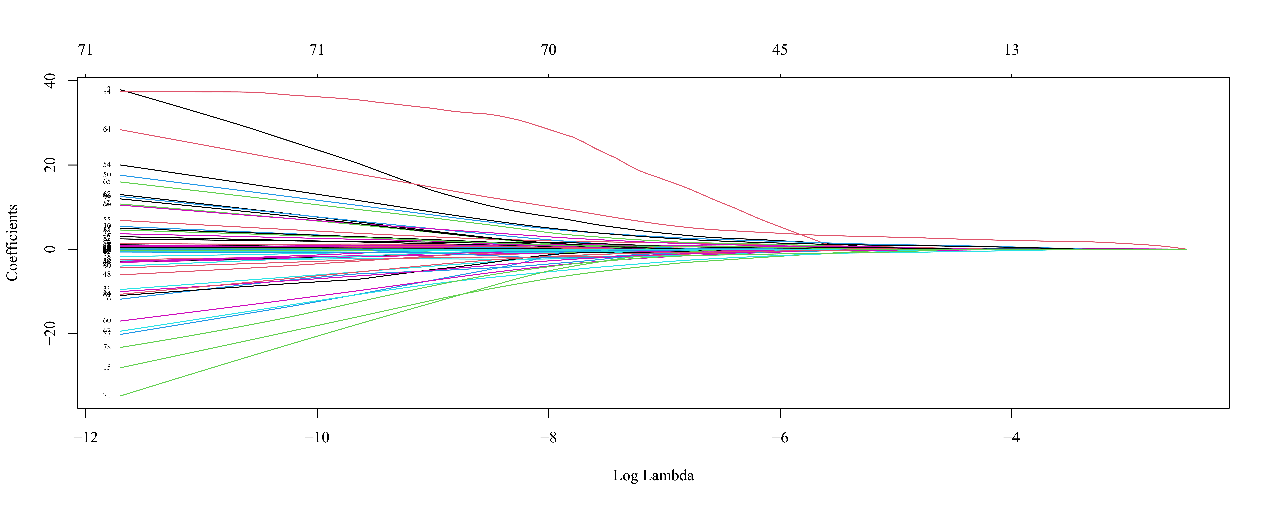


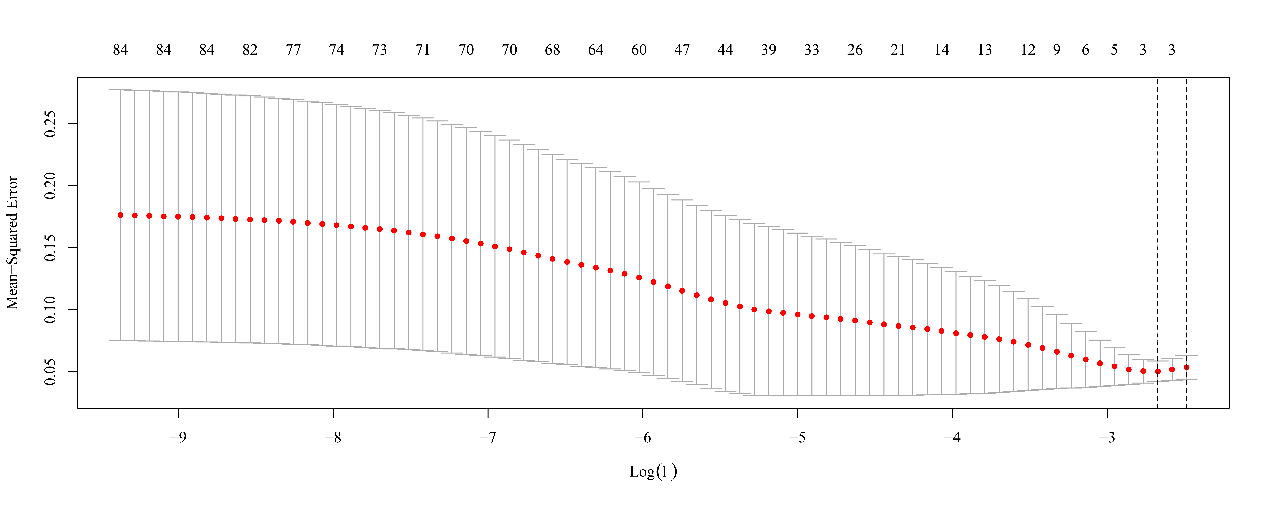


Figure S1. LASSO regression selection for MACE in training set.


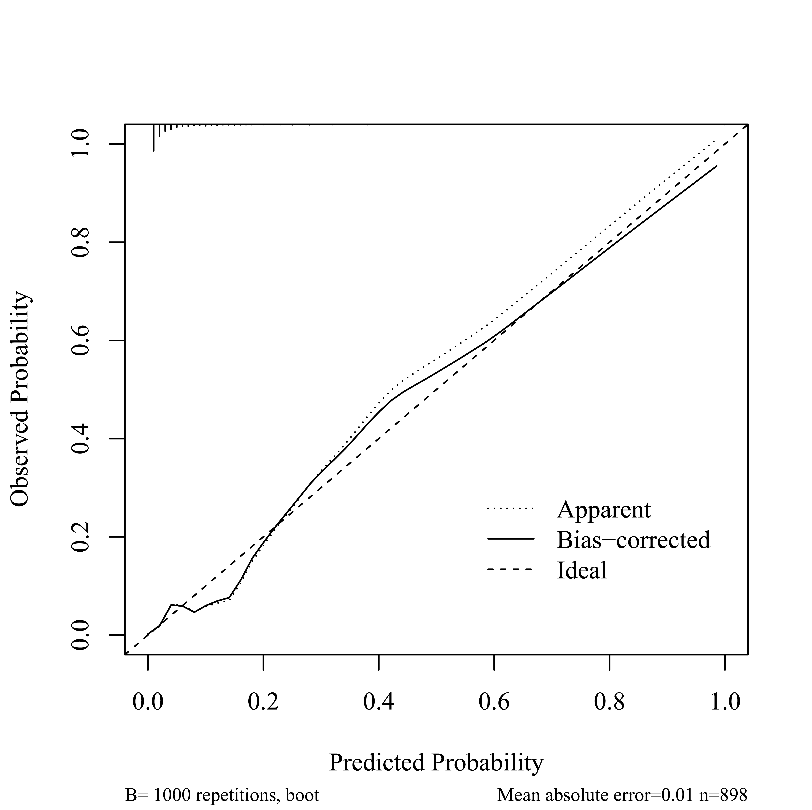


Figure S2. The calibration curve of nomogram in Wuhan subgroup.


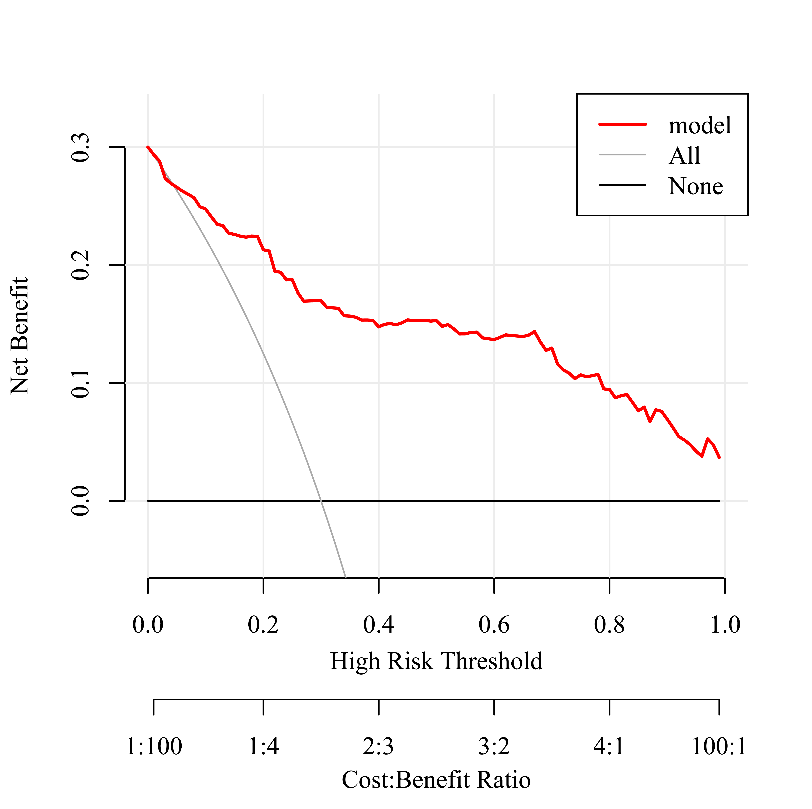


Figure S3. The DCA of nomogram in Wuhan subgroup. DCA：decision curve analysis


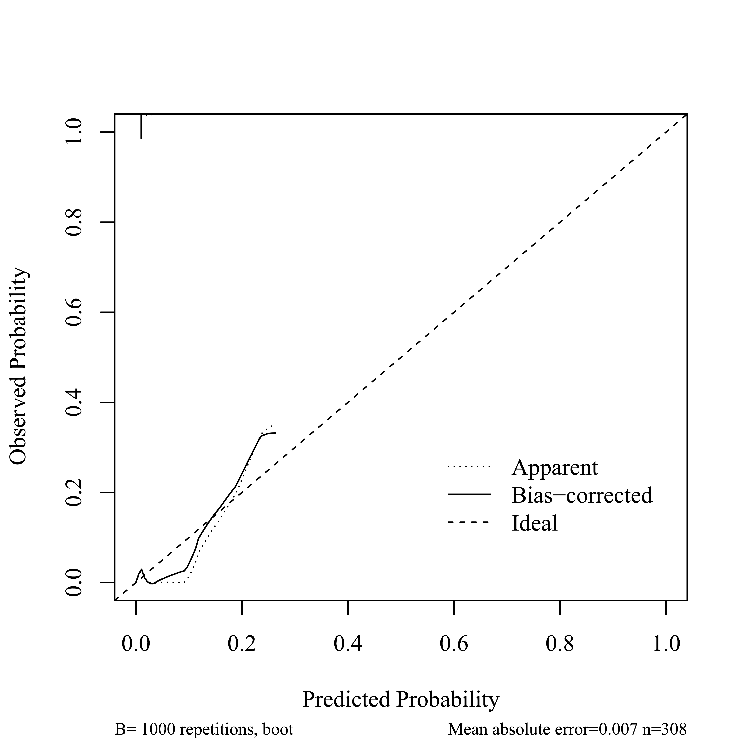


Figure S4. The calibration curve of nomogram in Sichuan subgroup.


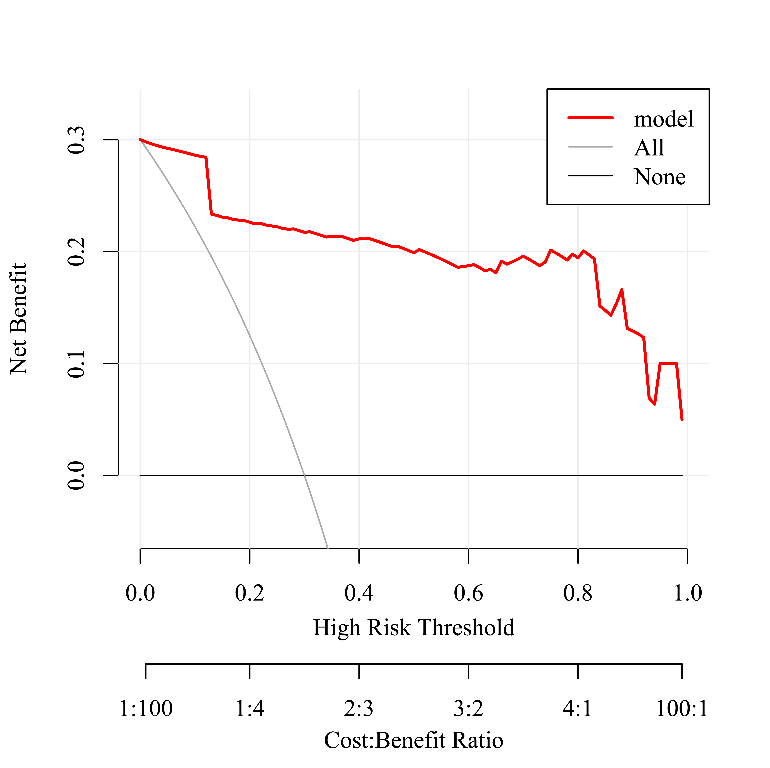


Figure S5. The DCA of nomogram in Sichuan subgroup. DCA：decision curve analysis
